# Commentary: Alexithymia, not autism, is associated with impaired interoception

**DOI:** 10.3389/fpsyg.2016.01103

**Published:** 2016-07-22

**Authors:** Lucy A. Livingston, Louise M. Livingston

**Affiliations:** ^1^MRC Social, Genetic and Developmental Psychiatry Centre, Institute of Psychiatry, Psychology and Neuroscience, King's College London, University of LondonLondon, UK; ^2^Department of Psychological Science, Birkbeck College, University of LondonLondon, UK

**Keywords:** autism, autism spectrum disorder, interoception, alexithymia, emotional symptoms

Alexithymia is characterized by difficulties in recognizing and reporting on one's own emotions (Nemiah et al., 1976). There is growing interest in alexithymia in Autism Spectrum Disorder (ASD) since at least 50% of those with ASD experience co-occurring alexithymia (Hill et al., 2004). Impaired interoception, that is problems with perceiving one's internal bodily signals, is a candidate mechanism underlying alexithymia (Herbert et al., 2011). However, interoceptive difficulties have also been implicated in ASD. It is therefore contentious whether alexithymia, ASD or both are linked to poor interoception (Brewer et al., 2015). A new study published in *Cortex* addresses this issue, concluding that alexithymia, not autism, is related to interoceptive impairment (Shah et al., 2016). In light of this, the role of alexithymia in emotional and interoceptive difficulties in ASD and arising clinical implications are discussed.

Bird and Cook's (2013) alexithymia hypothesis proposes that emotional problems observed in ASD are attributable to co-occurring alexithymia, rather than ASD itself. This arose from the findings that difficulties with empathy (Bird et al., 2010), emotional recognition (Cook et al., 2013) and attention to the eyes (Bird et al., 2011) are not associated with ASD after controlling for alexithymia. More recently, in elucidating the overlap between alexithymia and ASD, attention has turned to interoception. Quattrocki and Friston (2014) propose that early disturbance in oxytocin-mediated interoception promotes a developmental cascade of problems in representing emotional states, hence alexithymia. Although this model set out to explain all ASD symptomatology, Brewer et al. (2015) have extended the alexithymia hypothesis by arguing that where emotional symptoms are seen in ASD due to impaired interoception, these may be underpinned by co-occurring alexithymia.

Shah et al. (2016) quantified the distinct contributions of alexithymia and autism to interoceptive accuracy specifically, by instructing participants to count their own heartbeats within varying time frames, whilst actual heartbeat signals were recorded simultaneously. Across clinical and non-clinical populations, alexithymia, but not autism, was associated with poorer interoceptive accuracy. Crucially, the fact that individuals with and without ASD displayed comparable interoceptive accuracy when matched for alexithymia suggests that interoceptive atypicalities, when observed in ASD, are alexithymia-specific, which corroborates the alexithymia hypothesis. Furthermore, alexithymia predicted interoceptive accuracy over and above anxiety, depression and Body Mass Index, all of which may be related to interoception (Craig, 2014).

Shah and colleagues' findings add to growing evidence that the abilities to perceive your own emotions and bodily signals are tightly coupled (Seth, 2013). Indeed, an overlapping neural basis within the insula is implicated in both (Zaki et al., 2012). Therefore, interoception failure seems a reasonable mechanism underpinning alexithymia that warrants further investigation. Quattrocki and Friston (2014) offer a testable account of how perturbed oxytocin-mediated interoception might promote atypical social-emotional development. However, in light of Shah et al.'s findings, this perhaps should be recast as an account of alexithymia, rather than one capable of explaining all ASD-related phenomena.

Important implications for clinical research arise from Shah and colleagues' study. Their findings support the notion that an ASD diagnosis reflects a clustering of etiologically distinct symptoms (Happé et al., 2006). Therefore, co-occurring alexithymia, driven by poor interoception, may signify a particular subgroup of individuals with ASD who experience emotional as well as core autistic difficulties. This implies that managing the emotional difficulties in ASD may be possible without needing to “treat” the core features of ASD. Henceforth, amongst those with ASD and co-occurring alexithymia, interoceptive-based intervention (e.g., interoceptive training, endogenously-administered oxytocin) may be beneficial, for emotional symptoms at least. Although mixed findings are reported on the effectiveness of nasally-administered oxytocin in ASD (Bethlehem et al., 2014), crucially these studies neglect co-occurring alexithymia. Further, as Quirin et al. (2014) highlight, oxytocinergic intervention, which is proposed to enhance the integration of emotional information, may not be universally beneficial to all alexithymic individuals. Additional moderating factors, such as symptom severity and context, should be considered. As it stands, clinical awareness of co-occurring alexithymia in ASD is poor. Nonetheless, Samur et al. (2013) have outlined exciting opportunities for translating alexithymia research into useful clinical tools: Knowledge of alexithymia and interoception could be particularly useful for autistic individuals, their families and carers. For example, supporting people with ASD to be more aware of their internal bodily signals may be of therapeutic value, although further research on the efficacy of interoceptive training is required.

Other research questions concerning the links between ASD, alexithymia and interoception also warrant further investigation. Interoception might be separated into sub-components (see Garfinkel and Critchley, 2013) that differentially contribute to social and non-social symptoms in ASD. Future research should measure these sub-components, for example interoceptive awareness as well as accuracy, when comparing alexithymic and non-alexithymic individuals with ASD. Another key remaining question pertains to why alexithymia and ASD, despite their relative independence, co-occur so frequently. One possibility is that interoceptive impairments, although primarily implicated in alexithymia, may have downstream consequences for other social difficulties that characterize ASD. In clarifying the nature of the relationship between alexithymia and ASD, developmental investigations will prove useful. Surprisingly, only one study has addressed elevated alexithymia in children with ASD (Griffin et al., 2016) and another in adolescence (Milosavljevic et al., 2016). A longitudinal study is yet to be conducted. A developmental approach, as Quattrocki and Friston (2014) have proposed, would serve to clarify the causal role of early interoceptive impairment in alexithymia and emotional symptoms in ASD.

In summary, Shah and colleagues' study is the first to tease apart the inter-relations between alexithymia, ASD and interoception. In particular, difficulty in detecting one's own heartbeat is not a principle feature of ASD itself, but rather one of co-occurring alexithymia. Prospects for using interoceptive/emotional difficulties as a means of informing subgroup-specific clinical management of ASD are promising. Nonetheless, determining why alexithymia prevalence is particularly heightened in the ASD population continues to be a fundamental research priority for which a developmental approach will be required.

## Author contributions

LAL drafted the manuscript. Both authors gave final approval for publication and edited the manuscript.

### Conflict of interest statement

The authors declare that the research was conducted in the absence of any commercial or financial relationships that could be construed as a potential conflict of interest.
